# Development and Function of the Blood-Brain Barrier in the Context of Metabolic Control

**DOI:** 10.3389/fnins.2017.00224

**Published:** 2017-04-21

**Authors:** Roberta Haddad-Tóvolli, Nathalia R. V. Dragano, Albina F. S. Ramalho, Licio A. Velloso

**Affiliations:** Laboratory of Cell Signaling and Obesity and Comorbidities Research Center, Faculty of Medical Sciences, University of CampinasCampinas, Brazil

**Keywords:** blood-brain barrier, neurovascular unit, development, hypothalamus, inflammation, obesity

## Abstract

Under physiological conditions, the brain consumes over 20% of the whole body energy supply. The blood-brain barrier (BBB) allows dynamic interactions between blood capillaries and the neuronal network in order to provide an adequate control of molecules that are transported in and out of the brain. Alterations in the BBB structure and function affecting brain accessibility to nutrients and exit of toxins are found in a number of diseases, which in turn may disturb brain function and nutrient signaling. In this review we explore the major advances obtained in the understanding of the BBB development and how its structure impacts on function. Furthermore, we focus on the particularities of the barrier permeability in the hypothalamus, its role in metabolic control and the potential impact of hypothalamic BBB abnormities in metabolic related diseases.

## Introduction

Blood vessels transport and deliver nutrients to warrant organism function. In the brain, they specialized into a dynamic structure that provides a protective and homeostatic interface between the central nervous system (CNS) and the remainder of the body. In this sense, the blood-brain barrier (BBB) controls the passage of selected substances into the brain, while transporting toxic products back into circulation. This efficient permeable boundary allows maintenance of the homeostasis of the CNS milieu and correct function of brain circuits.

The functionality of the BBB depends on a strict architecture. The combination of non-fenestrated brain endothelial cells (BECs) lining the walls of the CNS blood vessels, together with pericytes, neurons and glia constitute the neurovascular unit (NVU) and confer integrity to the BBB.

In the ventromedial hypothalamus, the barrier has specialized to allow the dynamic passage of hormones and nutrients from the blood to the energy-sensing arcuate nucleus of the hypothalamus (ARC) and the export of newly synthesized hormones to the pituitary. At the level of the median eminence (ME), the barrier is formed by fenestrated capillaries that allow faster transport of substances into the nutrient-sensing hypothalamic nuclei lying adjacent to it. However, ME tanycytes, specialized radial glia cells lining the walls of the third ventricle, form a physical barrier to control the correct transport of nutrients and metabolic hormones into the brain parenchyma (Weindl and Joynt, 1973; Ganong, 2000; Mullier et al., 2010; Rodríguez et al., 2010). The metabolic state, as well as the consumption of saturated fatty acids, can damage this barrier and alter the nutrient sensing of tanycytes (Lee et al., 2012; Haan et al., 2013; Langlet et al., 2013b). Changes in the development of the BBB in the hypothalamus, especially in the vicinity of the ME, may predispose to obesity (Lee et al., 2012; Kim et al., 2016).

## BBB structure and function

In the last decade, there has been exponential progress in the understanding of how BBB structure and function work both in health and pathological conditions (Daneman and Prat, 2015; Banks, 2016). The first evidence for the existence of a barrier controlling the passage of substances from the circulating blood to and from the CNS dates back to 1885 when the German researcher Paul Ehrlich carried out experiments injecting Trypan Blue dye into the bloodstream of mice. Surprisingly, he noted that the dye could penetrate several tissues but not the brain and spinal cord (Ehrlich, 1885; **Figure 3B**). Years later, further studies performed by Goldmann (1909, 1913), Ehrlich's student, indicated that injection of the same dye directly into the brain showed the opposite as observed previously: the brains turned blue, whereas the peripheral tissues did not (**Figure 3B**). The term “blood-brain-barrier” was introduced by Lewandowsky (1900), based on experiments demonstrating that neurotoxic substances, e.g., cholic acids or sodium ferrocyanide, exhibited neurological symptoms only after intraventricular applications but not when injected into the bloodstream. Only in the late 1960s, with the development of electron microscopy, Reese and Karnovsky (1967) were able to visualize for the first time, at ultrastructural level, that the brain endothelial cells present unique cell-to-cell junctions, constituting a structural barrier that creates an almost impermeable frontier between the blood and CNS (Ribatti et al., 2006).

**Figure 1:**
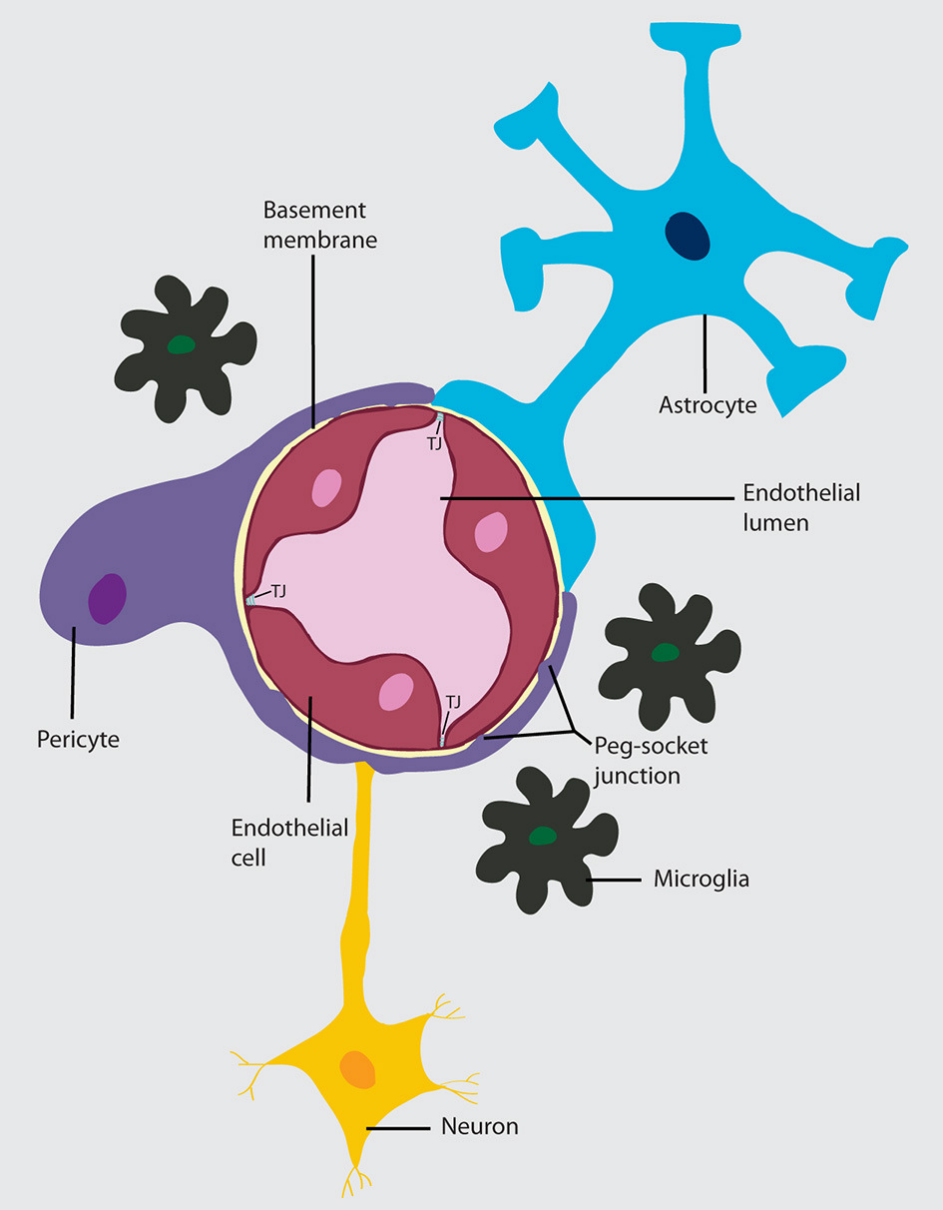
**Neurovascular unit (NVU)**. A layer of brain endothelial cells connected by tight junctions forms the blood-brain barrier. The intimate contact of these specialized endothelial cells with different cell types constitutes the NVU. A basement membrane embeds the brain endothelial cells, the pericytes, and astrocytes. In areas where the basement membrane is absent, brain endothelial cells and pericytes connect through peg-socket junctions. Astrocytes extend its end-feet and establish a close interaction with endothelial cells through transmembrane proteins, such as aquaporins. Astrocytes also connect with pericytes and neurons and together regulate BBB maintenance and function. The interaction of the cell components of the NVU with neurons and microglia can influence barrier function.

Anatomically, the BBB is comprised by a thin monolayer of BECs that are in intimate contact with vascular cells (pericytes and vascular smooth muscle cells), glial cells (astrocytes, microglia) and neurons. The crosstalk and molecular signaling between them are collectively known as the neurovascular unit (NUV) (Obermeier et al., 2013; Chow and Gu, 2015; Banks, 2016) (Figure 1). This close connection between different cells types within the NUV allows the BBB to properly perform its fundamental physiological functions.

Next, we present the main features of the cellular components of the BBB:

### Brain endothelial cells

To sustain a more restrictive permeability, BECs enclose specific barrier properties, which differentiate them from peripheral endothelial cells (Andreone et al., 2015; Chow and Gu, 2015). One particular feature is the presence of junction complexes between adjacent BECs involving transmembrane proteins and multiple cytoplasmic adaptor proteins located on the apical and lateral sides of the plasma membrane.

The structural integrity of BBB is sustained mainly by tight junction (TJ) proteins and *adherens* junctions (AJ). Specifically, TJ are composed of claudin family members, occludin, junctional adhesion molecules (JAMs) and *zonulae occludens* (ZO-1, ZO-2, and ZO-3)—membrane-associated accessory proteins that connect the cytoplasmic tails of claudins and occludin to the actin cytoskeleton to sustain the TJ structure (Luissint et al., 2012; Tietz and Engelhardt, 2015).

The primary biological role of TJs is to establish a rigorous restriction of paracellular molecular diffusion, creating a high electrical resistance between the endothelial cells and ions and other polar solutes. Brain endothelial cell's AJs, ubiquitous in the vasculature, consist of cadherin proteins extended throughout the intercellular cleft that mediate cell-to-cell membrane adhesion and are anchored into the actin cytoskeleton by the scaffolding proteins alpha, beta and gamma catenin (Abbott et al., 2010; Blanchette and Daneman, 2015).

Although, the specific AJs function in the BBB is yet to be fully elucidated, it is known that they play an important role in the maintenance of TJs and the junctional complex by keeping the BECs together (Keaney and Campbell, 2015). In addition, brain endothelial cells display low rates of transcellular vesicular transport in comparison to peripheral endothelial cells, a process termed transcytosis (transport between the luminal and abluminal cell membranes). Still, transcytosis is the most common mechanism to selectively uptake macromolecules such as albumin, low-density lipoprotein and hormones, e.g., insulin and leptin (Holly and Perks, 2006; Xiao and Gan, 2013; Chow and Gu, 2015). As a result of this restrictive barrier, BECs express several transporters and receptor proteins, as Glut-1, that selectively allow the entry of nutrients, neurotransmitters and other essential macromolecules into the brain parenchyma, at the same time that ensure the elimination of potentially metabolic waste and neurotoxic substances from the CNS into the blood (Keller, 2013; Keaney and Campbell, 2015).

Another important feature of the BECs is the low expression levels of leukocyte adhesion molecules (LAMs) such as E-selectin and Icam1, compared with ECs in non-neuronal tissues (Andreone et al., 2015; Daneman and Prat, 2015). This characteristic greatly restrains the number of immune cells that enter the CNS. In general, the physical barrier properties mediated by the junctional complex and the reduced transcytosis along with a highly selective cellular transport system illustrates how the brain endothelium orchestrate the regulation of the brain microenvironment.

### Pericytes

Pericytes were first characterized by Eberth (1871) and Rouget (1873) in the 1870's and initially named “Rouget cells.” Later, Zimmermann (1923) introduced the term pericyte due to their location in close proximity to the endothelial cells. Anatomically, pericytes are located at the abluminal surface of microvessels, embedded in a common basement membrane with the BECs, and exhibit elongated cytoplasmic processes that wrap the endothelium around (Trost et al., 2016). The CNS vasculature has significantly higher pericyte density/coverage compared with peripheral tissues, which correlates with the particular endothelial barrier properties. It is estimated that the ratio of pericytes to endothelial cells in CNS is 1:1-1:3, while this ratio appears to be from 1:10-1:100 in striated muscles (Shepro and Morel, 1993; Millis et al., 2013). Despite being separated by the basal membrane, in areas lacking a basement membrane (BM) BM, pericytes and BECs make direct peg-and-socket contacts, in which pericyte cytoplasmic fingers (pegs) are inserted into endothelial invaginations (pockets) (Winkler et al., 2011). Several different transmembrane junctional proteins are present at these contact points, including N-cadherin, forming key AJs between the two cell types; connexin-43 (CX43), hemichannels that form gap junctions enabling the exchange of nutrients, secondary messengers and ions; adhesion plaques composed predominately of fibronectin that anchor pericytes to endothelial cells (Berger et al., 2005; Zlokovic, 2008; Armulik et al., 2011; Winkler et al., 2011).

Pericytes play essential roles in the regulation of several neurovascular functions. They are critical during angiogenesis, participating in vessel formation, remodeling and stabilization (see developmental section) (Armulik et al., 2011; Winkler et al., 2011). *In situ* and *In vivo* studies evidenced that pericytes are modulators of capillary diameter and blood flow in response to changes in neural activity (Peppiatt et al., 2006; Fernández-Klett et al., 2010; Hall et al., 2014). However, Hill et al. (2015) demonstrate that capillary pericytes are not contractile *in vivo*. Thus, the active regulation of the capillary blood flow by pericytes remains controversial presumably due to the lack of proper pericyte definition and identification (Trost et al., 2016).

Apart of their importance in BBB development (see BBB development section), pericytes are involved in the formation and maintenance of the highly selected permeability of the BBB in adulthood and aging (Armulik et al., 2010; Bell et al., 2010; Daneman et al., 2010a). Pericyte deficiency promotes severe BBB dysfunction as a result of increased vascular permeability (directly correlated with absolute pericyte coverage, increased rates of transcytosis and paracellular transport due to reduced expression of various TJ and AJ proteins). Pericytes also guide astrocytic foot processes to surrounding CNS blood vessels and mediate the polarization of astrocytic end-feet, highlighting the interdependence among components of the NVU (Armulik et al., 2010; Keaney and Campbell, 2015).

Increasing evidence has shown that pericytes may constitute multipotent stem/progenitor cells that could differentiate into mesenchymal cells and into non-mesenchymal cell types, including glial and neuronal lineages under different *in vitro* conditions (Bribair et al., 2013). Until now, there is still a lack of studies describing that such differentiation occurs in the brain (Dore-Duffy and Cleary, 2011; Trost et al., 2016). Moreover, pericytes can control several aspects of the CNS immune response (reviewed in Rustenhoven et al., 2016).

### Astrocytes

Due to the position of astrocytes in the NUV (ensheathing almost 90% of brain microvasculature), many studies have focused on the role of astrocytes in the maturation and maintenance of BBB (Correale and Villa, 2009). Indeed, astrocytes can induce barrier properties in non-CNS endothelial cells *in vivo* (Janzer and Raff, 1987).

Astrocytes, the most abundant cells in the brain, are spongiform-shaped glial cells that extend many branching cellular processes, including astrocytic end-feet that cover brain endothelial cells. Specifically, astrocytic end-feets establish a close interaction with BECs through transmembrane proteins anchoring, such as the water channel Aquaporin-4 (Aqp4) and the potassium channel KIR4.1, critical for CNS water homeostasis regulation (Cabezas et al., 2014). Furthermore, astrocytes communicate between each other through gap junctions forming an extensive glial syncytium that is associated with well-coordinated responses within large groups of cells (Theis et al., 2005; Alvarez et al., 2013). Astrocytes perform several functions critical to CNS physiology, including the regulation of blood flow in response to changes in neuronal activity which results in enhanced delivery of oxygen and glucose to the active brain region; the maintenance of ion and neurotransmitters concentration within the extracellular space for proper synaptic transmission; the modulation of synaptic and neural activity by the release of gliotransmitters, such as glutamate, ATP, D-serine, and GABA; and are also able to respond to local levels of nutrients, glucose uptake from blood vessels and the storage of glycogen, which is hydrolyzed to release lactate into the extracellular space when glucose is scarce (Sofroniew and Vinters, 2010; Cabezas et al., 2014).

One of the essential roles attributed to astrocytes is to regulate the nutrient availability, such as glucose, and also the access of some peripheral hormones, including leptin, ghrelin and GLP-1. Their privileged anatomical position, in close proximity to the blood vessels and neurons, allows them to act as important metabolic sensors. Actually, hypothalamic astrocytes express specific transporters and receptors that are involved in the control of energy homeostasis. The astrocytic end-feet that enclose capillaries express the glucose transporter GLUT-1 that transports glucose into the CNS. The uptaken glucose is stored in form of glycogen, which is mobilized to release lactate to the neurons when glucose is not abundant, as in periods of energy deficit (Argente-Arizón et al., 2015; Chowen et al., 2016). Astrocytes also express the glucose transporter GLUT-2 that is important for glucosensing process and in the homeostatic control of circulating glucose levels and food intake (Marty et al., 2005; Stolarczyk et al., 2010). GLUT-2 inactivation results in overeating, providing evidence that glucose detection by GLUT2 contributes to the control of food intake by the hypothalamus (Bady et al., 2006; Stolarczyk et al., 2010). Leptin receptors are expressed in hypothalamic astrocytes and their physiological relevance was recently demonstrated. The conditional deletion of the leptin receptor in GFAP positive cells in mice decreased the number and length of astrocyte projections in hypothalamic neurons involved in feeding control, such as POMC and AGRP neurons of the arcuate nucleus. In addition, leptin-regulated feeding was diminished in mice with astrocyte-specific leptin receptor deficiency (Kim et al., 2014).

Regarding the contribution of astrocytes in BBB development, they do not appear to participate in this process at early embryonic stages (see development section) (Daneman et al., 2010b). Indeed, mature astrocytes produce factors that regulate BBB function and integrity. Astrocytes also secrete angiopoietin-1 and angiotensin that restrict BBB permeability by supporting efficient organization of TJs (Lee et al., 2003; Wosik et al., 2007; Siegenthaler et al., 2013). Thus, astrocytes are critical in sustaining BBB functionality and integrity as well as in the regulation of neuronal and synaptic activity.

### Microglia

In addition to pericytes and astrocytes, the brain endothelium at the NUV is in close contact with immune cells. The two main immune cell populations within the CNS are perivascular macrophages and microglia.

Perivascular macrophages are monocytic lineage cells located outside of the CNS parenchyma. In fact, these cells reside in and circulate through the Virchow–Robin spaces, which lie between the BM around pericytes and at the surface of the glia interface of the brain vessels. The perivascular macrophage number is continuously maintained by replacement from blood-borne cells macrophages. They provide a first line of innate immunity due to their ability to quickly phagocytose particles from the cerebrospinal fluid (Daneman and Prat, 2015).

The brain parenchyma is populated by microglia, the most abundant CNS innate immune cells. Microglial cells, also referred as resident macrophages of the brain, are derived from progenitors in the yolk sac, which migrate into the CNS parenchyma during neonatal development. Microglia colonization comes first during BECs invasion into the brain (da Fonseca et al., 2014) and are shown to be involved in CNS vasculogenesis, promoting endothelial cell fusion to increase vascular complexity (Fantin et al., 2010), as well as decreasing paracellular permeability in cultured BECs (Zenker et al., 2003). Accordingly, these data suggest that the interactions between microglia and the brain endothelium could participate in the BBB formation and regulation (Correale and Villa, 2009; da Fonseca et al., 2014).

Generally, microglia displays two distinct morphological patterns. The steady-state population of microglia in the healthy brain exhibits a 'resting' phenotype, which is characterized by a small, and circular cell body with extensively ramified processes. As a part of their homeostatic functions, microglial cell bodies remain stationary, but their processes continuously scan the surrounding extracellular space and communicate directly with neurons, astrocytes, and blood vessels (Nayak et al., 2014). Microglia rapidly respond to injury signals or infection through an activated phenotype characterized by a morphological change into an amoeboid shape and alterations in signaling and gene expression in order to perform inflammatory functions (Lull and Block, 2010; Saijo and Glass, 2011).

Prolonged microglia activation and consequent chronic neuroinflammatory state in CNS might induce impairments in BBB integrity that, in turn, contribute to the progression of neurodegenerative diseases (reviewed in de Vries et al., 2012). Evidence from *in vitro* studies has shown that microglia activation may be related to BBB disruption. Sumi et al. (2010) demonstrated that the treatment of a rat brain microvascular endothelial cell/microglia co-culture system with lipopolysaccharide, a microglial activator, induced an increase in endothelial cell permeability and changes in the expression pattern of tight junction proteins. Another study using a similar *in vitro* co-culture system found that the tumoral necrosis factor α (TNF-α) released from activated microglia also increased the permeability of BECs, which could be blocked by a neutralizing antibody against TNF-α (Nishioku et al., 2010).

Although, these *in vitro* BBB models are important for unraveling the mechanisms involved in the crosstalk between microglia activation and BBB integrity, more studies are needed to improve our understanding on how these cell types interact *in vivo*.

## BBB development

**Figure 2:**
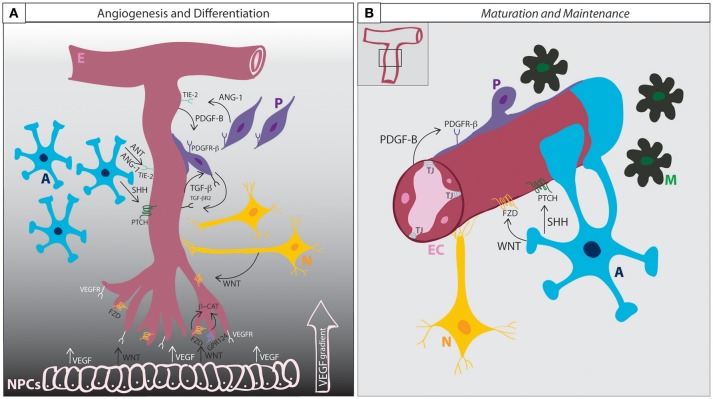
**BBB development and maintenance**. **(A)** Angiogenesis and differentiation. VEGF secretion from neural progenitor cells induces endothelial cells penetration into the brain parenchyma according to a VEGF concentration gradient. Wnt ligands also secreted from the NPCs induces the migration of the endothelial cells and activate β-catenin signaling through the binding to Frizzled receptors, inducing the expression of BBB specific genes. GPR124, together with WNT, co-activates β-catenin signaling. Endothelial cells secrete PDGF-B and attract pericytes expressing PDGFR-β. The interaction between ECs and Ps induce the mutual expression of TGF-β and TGF-βR2. The activation of TGF-β signaling regulates basement membrane formation and the induction of Ang-1 expression in pericytes, that acting through the endothelial receptor Tie-2, enhance tight junction expression. Astrocytes release SHH, that when bound to PTCH receptor induces Shh signaling activation in ECs and contributes to BBB formation. Astrocytes also express ANT and ANG-1, which by limiting BBB permeability contributes to the maturation of BBB function. **(B)** Maturation and Maintenance. Pericytes and astrocytic end-feet cover the endothelium and secrete matrix proteins that will constitute the basement membrane. Astrocytes continue secreting SHH and WNT in order to maintain BBB functionality throughout life. A, astrocytes; E, endothelium; EC, endothelial cell; M, microglia; N, neuron; NPC, neural progenitor cell; P, pericyte; TJ, tight junction; Ang-1, angiopoietin-1; ANT, angiotensin; β-cat, beta-catenin; VEGF, vascular endothelial growth factor; VEGFR, vascular endothelial growth factor receptor; FZD, frizzled; GPR124, adhesion G protein coupled receptor A2; Shh, sonic hedgehog; Ptch, Patched 1; PDGF-B, platelet derived growth factor B; PDGFR-β, platelet derived growth factor receptor beta; TGF-β, transforming growth factor-beta; TGF-βR2, transforming growth factor beta receptor type-2.

The development and differentiation of the BBB can be subdivided into three phases: i, angiogenesis; ii, differentiation; and iii, maturation (summarized in Figure 2).

The angiogenic phase begins early during neural tube development (as early as E9.0-E10.5 in mice). Endothelial cells from the perineural vascular plexus penetrate the neuroectoderm according to a vascular endothelial growth factor (VEGF) concentration gradient giving rise to immature brain vessels (Raab et al., 2004; Potente et al., 2011). VEGF is expressed by the neural progenitors of the ventricular neuroepithelium and serves, together with angiopoietin, as a major driving force for the migrating endothelial cells (EC) (Risau et al., 1986). VEGF deficiency is lethal (Shalaby et al., 1995; Carmeliet et al., 1996). Downstream VEGF signaling supports angiogenesis through endothelial cell proliferation, migration and survival (Olsson et al., 2006). Together with VEGF, neural Wnt signaling plays an important role in the development of the BBB (Daneman et al., 2009). Different Wnt ligands, including Wnt7a, Wnt7b, and Wnt3a are secreted by the neuroepithelium (Wang et al., 2012) and induce further ingression of ECs into the neural tissue, activating Wnt/B-catenin signaling in the newborn endothelial cells (Liebner et al., 2008; Daneman et al., 2009; Zhou and Nathans, 2014) that leads to induction of genes critical for the BBB formation and vascular patterning, such as glucose transporter Glut-1 (Stenman et al., 2008), death receptors DR6 and TROY (Tam et al., 2012) and tight junction proteins. Defects in Wnt/β-catenin signaling result in major vascular malformations and BBB breakdown (Stenman et al., 2008; Daneman et al., 2009; Wang et al., 2012). Retinoic acid, Notch signaling receptor tyrosine kinase, cadherins and ephrins also contribute to angiogenesis in the CNS (Adams and Alitalo, 2007; Dejana and Vestweber, 2013).

At about E15.5, the newly formed vessels continue to differentiate and mature as a complete NVU forming the basis for the BBB. During the differentiation phase (between E15.5 and E18.5 in mice), the brain barrier is structured properly by the induction of anti-angiogenic signals and the recruitment of pericytes and astrocytes to the newly formed vessels. Pericytes express the platelet-derived growth factor receptor- β (Pdgfr-β) and are directed to the endothelial developing capillaries that secrete Pdgf-B (Lindahl et al., 1997; Hellstrom et al., 1999). Pdgfr-β mouse mutants completely lack brain pericytes, and die as consequence of brain microhemorrhages (Lindahl et al., 1997; Lindblom et al., 2003; Tallquist et al., 2003); thus, pericyte recruitment to the developing endothelial capillaries is critical for the formation and maintenance of the BBB (Armulik et al., 2010; Bell et al., 2010; Daneman et al., 2010a). The lack of Pdgf-β or Pdgfr-β leads to erroneous TJ distribution and increased vascular permeability (Hellstrom et al., 2001).

Interactions between pericytes and the brain endothelial cells lead to the expression of transforming growth factor-β (Tgf-β) and its receptor (Tgf-βR2) by both cell types. TGF secretion induces cell adhesion through the production of cadherin-2 by the endothelial cells and the secretion of different extracellular matrix components that contribute to basement membrane (BM) formation by the pericytes (Winkler et al., 2011). Notch and sphingosine-1-phosphate (S1P) signaling also contribute to the regulation of cadherin-2 expression in BECs (Winkler et al., 2011; Obermeier et al., 2013). Upon activation of Tgf-β signaling, pericytes produce Ang-1 that, by enhancing tight junctions formation, limit BBB permeability, and reduce the expression of leukocyte adhesion molecules (LAMs). Pdgf-β and Wnt/β-catenin signaling is also important during the differentiation of the BBB by the induction of transporters and increased expression of tight junctions. This provides integrity to the barrier (Liebner et al., 2008; Daneman et al., 2009; Tam et al., 2012; Wang et al., 2012; Zhou and Nathans, 2014; Andreone et al., 2015). In the BBB, Gpr124 has been described as a necessary endothelial receptor specifically in the CNS and function as a co-activator of Wnt/b-catenin signaling to mature the BBB (Kuhnert et al., 2010; Cullen et al., 2011; Zhou and Nathans, 2014).

Pericytes are apparently required to guide astrocytes toward the developing BBB (Armulik et al., 2010). Once recruited to the forming NVU, astrocytes are involved in limiting BBB permeability by the release of Sonic Hedgehog (Shh). The activation of Shh signaling leads to the expression of occludin and claudin5 and inhibition of chemokines and cell adhesion molecules in the endothelial cells, suggesting a role for astrocytes and Shh in maintenance of BBB functionality and immune surveillance (Alvarez et al., 2011; Obermeier et al., 2013; Siegenthaler et al., 2013). Recent studies have uncovered yet another important role for astrocytes in the production of retinoic acid, which is also necessary for the correct development of BBB vessels (Halilagic et al., 2007; Mizee et al., 2013).

Maturation and maintenance of the BBB is achieved by the persistence of tight junction proteins expression and their redistribution throughout the whole BBB structure. The production of TJs is regulated by Wnt signaling between astrocytes and BECs. Close contact between the endothelial cells, pericytes, astrocytes, and possibly neurons and microglia sustain BBB integrity and function as a stabilized neurovascular unit (reviewed in Obermeier et al., 2013; reviewed in Engelhardt and Liebner, 2014; reviewed in Zhao et al., 2015). The BBB is already formed and completely functional during late gestation in rodents and in the third trimester in humans (Bauer et al., 1995; Daneman et al., 2010a; Ben-Zvi et al., 2014). In the rat embryo, the BBB is already functional at E16 (Saunders et al., 2012). The exact time window remains elusive and is likely species dependent (Saunders et al., 2013; Hagan and Ben-Zvi, 2015).

There has been intense debate on the BBB features that provide its correct function. To date, it is believed that tight junctions are functional in sealing the space between BBB endothelial cells very early in development (Dziegielewska et al., 1979; Bondjers et al., 2006; Tam et al., 2012; Ben-Zvi et al., 2014) but only when the intracellular pathway of transcytosis is partially downregulated will the complete restrictive properties of the barrier become fully matured (Hagan and Ben-Zvi, 2015). Little is known about the functionality of transporters at the embryonic BBB, although some transporters, like Glut-1, are known to be expressed very early (Boado and Pardridge, 1990; Farrell and Pardridge, 1991; Bauer et al., 1995; Hagan and Ben-Zvi, 2015). A role of Mfsd2a in participating in the inhibition of vesicular trafficking and inducing a more sealed barrier during development was suggested recently (Ben-Zvi et al., 2014; reviewed in Hagan and Ben-Zvi, 2015). Mfsd2a mutants have a defective BBB even though presenting proper and functional tight junctions (Ben-Zvi et al., 2014). Thus, barrier transporting properties would be determined very early in development while the sealing function would be acquired gradually across development, first with the suppression of fenestrations, then with the appearance of functional TJs, and finally with the decrease of transcytosis by the expression of Mfsd2 (Ben-Zvi et al., 2014; Hagan and Ben-Zvi, 2015).

Alterations in barrier development and in TJ expression could lead to anomalies later in life as well as to increased predisposition to develop metabolic diseases. Maternal obesity increases BBB permeability in the offspring (Kim et al., 2016), leading to higher exposure to leptin and ghrelin. In consistence with that, overnutrition during early postnatal life alters brain sensitivity to ghrelin (Collden et al., 2014), suggesting that nutrient sensing control alterations during specific hypothalamic developmental time-points could contribute to metabolic defects in the adult life. These alterations can affect the programming of energy homeostasis circuits predisposing the offspring to the development of metabolic syndrome at early life and/or adulthood.

## BBB in the hypothalamic area

In some areas of the brain, the BBB is modified in order to allow the access of certain substances from systemic circulation into the central nervous system. This occurs particularly in periventricular areas creating a blood/spinal fluid interface (Bennett et al., 2009). In these regions, the BBB capillaries are highly fenestrated with less tight junctions between endothelial cells creating a more permeable barrier (Bennett et al., 2009). Seven periventricular regions display differential barrier properties and are collectively known as the circumventricular organs: i, sub-fornical organ; ii, organum vasculosum of the lamina terminalis; iii, pituitary; iv, area postrema; v, median eminence (ME); vi, subcomissural organ; and vii, pineal (Bennett et al., 2009; Szathmari et al., 2013). In these regions, in addition to the cells that classically form the BBB vessels, a differential type of radial glial cell, termed tanycytes are also found in the interface between the spinal fluid and the capillaries (Rodríguez et al., 2010). Because of its physical proximity with the hypothalamus, the BBB at the ME is of particular interest regarding whole-body energy homeostasis and metabolism.

The median eminence is adjacent to the arcuate nucleus (ARC) of the hypothalamus. This region harbors neurons that play important roles in the control of whole body metabolism (Velloso and Schwartz, 2011; Cavadas et al., 2016). Classically, two main neuronal populations control energy homeostasis in the ARC: i, NPY/AgRP neurons, which are active during fasting and provide orexigenic and anti-thermogenic signals; ii, POMC/CART neurons, which are active following food intake and provide anorexigenic and pro-thermogenic signals (Velloso and Schwartz, 2011; Cavadas et al., 2016). In addition, other distinct hypothalamic neuronal subpopulations have been described recently (Zhang and van den Pol, 2016; Campbell et al., 2017; Fenselau et al., 2017; Lam et al., 2017). Of note, a recent study has identified ARC neurons that express the dopamine-synthesizing enzyme, tyrosine hydroxylase (TH). These cells are involved in the integration of homeostatic and hedonic feeding signals and are potential targets for the treatment of obesity (Zhang and van den Pol, 2016).

Because ARC neurons are involved in the sensing of the nutritional status of the body, it would be expected that they were located in an anatomical area where the access to nutrients and energy-status signaling substances would be facilitated. In fact, studies have shown that the interface between the median eminence and the ARC is somewhat leaky to hormones and nutrients (Obici and Rossetti, 2003; Lam et al., 2005). For example, fatty acids present in the systemic circulation are not freely diffusible to most areas of the CNS (Mitchell and Hatch, 2011). In general, they rely on particular transport systems present in the structures of the BBB (Betsholtz, 2014). The proper function and distribution of these transport systems is of major importance in the development and physiology of the brain throughout life because most fatty acids that comprise the central nervous system phospholipids cannot be synthesized *de novo* in the brain, and thus, must be obtained from the systemic circulation (Mitchell and Hatch, 2011). However, in the ME specific conditions make circulating fatty acids more available to ARC neurons (Obici and Rossetti, 2003; Lam et al., 2005).

As early as the 1970's, studies have revealed that hormones involved in energy homeostasis and nutrients have particular properties to cross the BBB at the ME/ARC interface. Systemic insulin crosses the BBB by a saturable system (Woods and Porte, 1977) and concentrates mostly in the olfactory bulb and hypothalamus (Havrankova et al., 1981). Similarly, leptin reaches the brain by a saturable system, which is independent of insulin and is highly detectable in the choroid plexus, the ME and the ARC (Banks et al., 1996). A major advance in understanding how hypothalamic neurons are exposed to hormones and nutrients to respond to systemic variations in whole body energy status was obtained by the characterization of the presence of nutrient transporters in BBB endothelial cells. Beginning in the 1990's a series of studies have explored the particularities of how peptides can cross the BBB (Cashion et al., 1996; Pam et al., 1996; Banks et al., 1997). The BBB endothelial cells are also provided with transport systems for glucose (Pardridge et al., 1990); fatty acids (Obici and Rossetti, 2003; Lam et al., 2005) and aminoacids (Hawkins et al., 2006), and the variations in whole body energy status, such as in fasting/feeding or metabolic diseases can affect function of such transport systems (Kastin and Akerstrom, 2000; Kastin et al., 2001). This implies that the BBB at the ME/ARC interface plays a major role in controlling the way hypothalamic neurons are exposed to systemic factors involved in metabolism and nutrition. In this context, important advance in the field by exploring the mechanism involved in the regulation of the ME tanycytes has been provided over the last few years.

**Figure 3:**
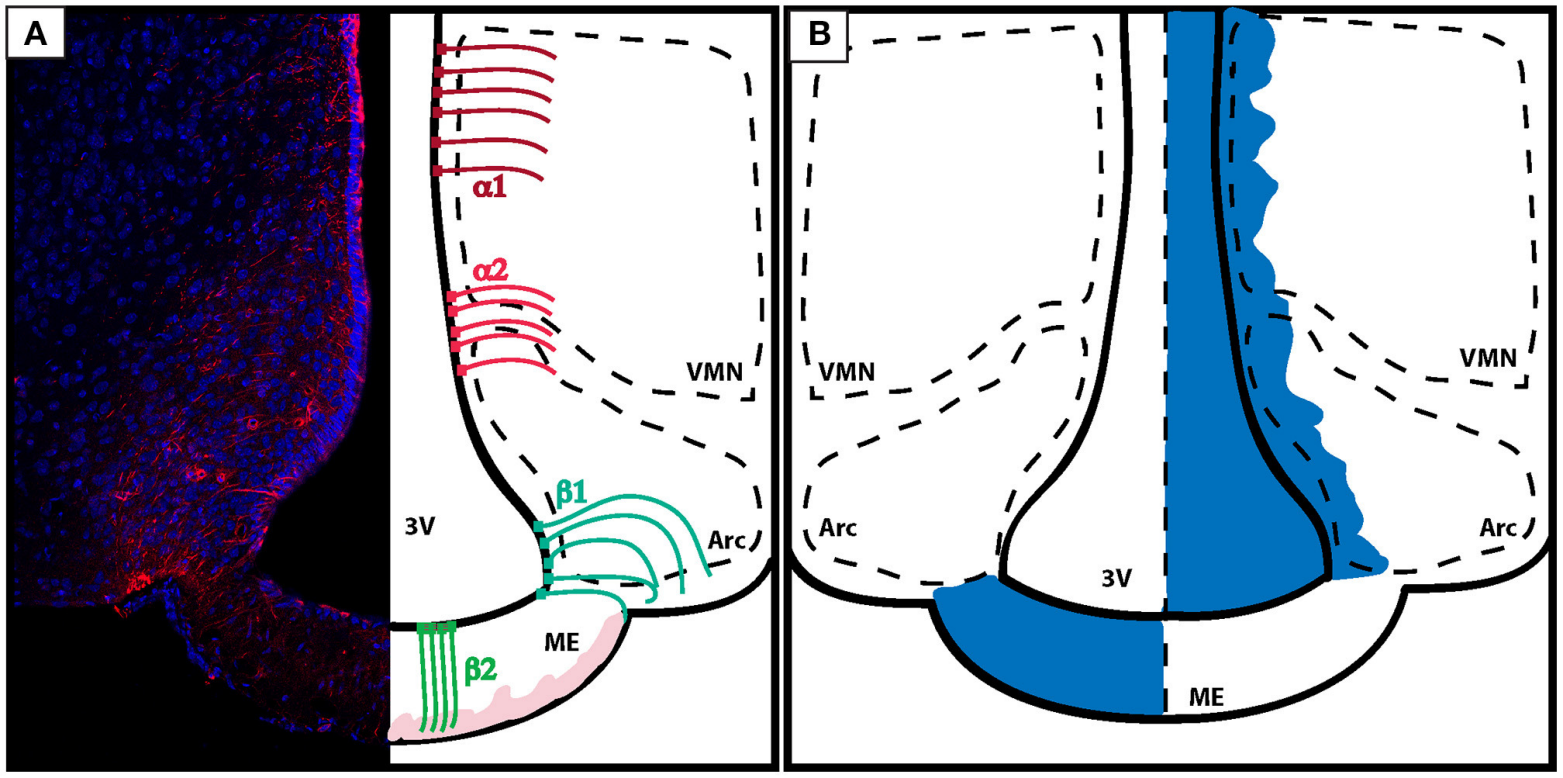
**Organization of the BBB in the energy-sensing hypothalamus. (A)** Coronal section of the tuberal hypothalamus showing the distribution of tanycytes along the third ventricle wall. Left: vimentin staining (red) shows the projections of tanycytes to the brain parenchyma. Right: tanycytes line the third ventricle and can be classified according to location and function. α tanycytes don't possess barrier properties. α1 tanycytes (dark red) lye in the dorsal ventro-medial nucleus of the hypothalamus, while α2 tanycytes (light red) are found in between the ARC and the VMH. β tanycytes are located ventrally and function as gatekeeper cells controlling the passage of substances from the leaky ME to the ARC. β1 tanycytes (turquose) divide the ME from the ARC, while β2 tanycytes (green) are located in the ME, and characterized by processes with direct access to the blood capillaries. Blood capillaries are displayed in light pink. **(B)** Diagram showing how the diffusion of dyes injected peripherally do not penetrate the brain (exemplified here by the action of tanycytes lining the ME and the ARC) (left). On the other hand, dyes that are infused inside of the brain ventricles diffuse trhough the CSF and penetrate the brain parenchyma but do not pass the ME in the direction of the portal capillaries (right). VMN, ventro-medial nucleus; ARC, arcuate nucleus; ME, median eminence; 3V, third ventricle.

Tanycytes are originated from the radial bipolar glia around E17.5 in mice and have a morphology that is somewhere in between the aspects of an ependymal cell and an astrocyte; differing from the last by having a single basal projection directed toward the parenchyma of the brain (Rodríguez et al., 2010; Figure 3A).

Mounting evidence suggests that, at least part of the selective leakiness of the BBB at the median eminence relies on the responsiveness of tanycytes to nutrients present in the bloodstream. Early studies evaluating hypothalamic tanycytes have demonstrated their role in the transport of substances between the third ventricle and ME (Wagner and Pilgrim, 1974). Also, tanycytes were shown to respond to hormone production and direct hormone delivery to certain anatomical sites and systemic circulation (Akmayev and Popov, 1977; Vallet et al., 1991). Interestingly, tanycytes respond rapidly to systemic stimulation, suggesting that this particular cell-type could play a role in the dynamic control of median eminence and adjacent areas exposure to systemic factors (Lichtensteiger et al., 1978). Recent studies have provided important progress in the understanding of the roles played by tanycytes in the ME and ARC (Langlet et al., 2013a,b).

The identification of leptin in the mid 1990's placed the ARC in the center of a complex system that controls body energy status (Zhang et al., 1994). With this concept in mind, researchers looked for a potential involvement of ME tanycytes as gatekeepers controlling the access of substances (particularly nutrients and hormones) that could modulate ARC-neuron function. However, it was only in 2011 that first evidence was provided showing that tanycytes are responsive to glucose fluctuations promoted by feeding (Frayling et al., 2011). Glucose leads to a powerful ATP-mediated Ca^2+^ release into the tanycytes, which in turn release ATP to adjacent cells. Therefore, it was proposed that by responding to glucose and releasing ATP, ME tanycytes could modulate the activity of ARC neurons (Frayling et al., 2011). Furthermore, ME tanycytes can transport leptin into the mediobasal hypothalamus (Balland et al., 2014). When leptin present in the bloodstream reaches the median eminence it activates leptin receptors expressed in the tanycytes. This in turn, activates the protein ERK that transduces the signals required for an appropriate transport of leptin through the cerebrospinal fluid into the ARC (Balland et al., 2014).

Interestingly, in order to respond to the constant changes in nutrient and hormone availability in the circulation, the plasticity of the median eminence tanycytes has proven remarkable. During the physiological cycles of fasting and feeding these cells undergo both morphological and functional changes (Langlet et al., 2013a). The decreased blood levels of glucose during fasting are capable of inducing changes in the structure of the interface between the blood and the ARC. At least in part, these morphological and functional changes are dependent of the expression of VEGF-A by the tanycytes (Langlet et al., 2013a).

In addition to glucose and leptin, fatty acids can also affect ME tanycytes. In diet-induced obesity, the amount of lipid droplets increase considerably in tanycytes (Hofmann et al., 2017). Moreover, the ratio between saturated and unsaturated lipids is modified suggesting that this cell type may also act as a sensor and gatekeeper for lipids in the median eminence/ARC interface (Hofmann et al., 2017).

## Concluding remarks

The BBB provides a physiological interface between the central nervous system and the systemic circulation allowing the entrance of nutrients and certain signaling molecules and restraining the entrance of microorganisms and particles that can harm the brain. Because hypothalamic neurons must sense the energy status of the body, the BBB at the median eminence specialized to a more permeable interface between the vasculature and the brain. Recent studies have demonstrated that tanycytes present in the interface between the ARC and the median eminence are very sensitive to nutrients. They can be rapidly modified in response to fast and fed states and also, can be disturbed by abnormal consumption of nutrients derived from the diet. In obesity and in metabolic conditions associated with the obese phenotype, hypothalamic neurons are affected by a local inflammatory response that is triggered by the excessive amount of fatty acids in the diet. Mounting evidence suggest that, at least in part, the anomalous activity of the hypothalamic tanycytes can play a role in the defective neuronal activity in obesity and associated conditions. Future studies should focus on the identification of mechanisms that may protect the tanycytes from diet-induced abnormalities and the impact of such protection in the progression of metabolic diseases.

## Author contributions

RHT, NRVD, and LAV discussed the structure of the manuscript. NRVD wrote the aspects of the BBB structure and function and RHT wrote the developmental topic. LAV and AFSR wrote BBB specialization in the hypothalamus. RHT, NRVD, and LAV wrote the introduction, abstract, and conclusion remarks. RHT made the figures and figure legends.

### Conflict of interest statement

The authors declare that the research was conducted in the absence of any commercial or financial relationships that could be construed as a potential conflict of interest.
